# Real-World Insights Into Hypoglycemia Risk While Driving in Teens and Young Adults With Type 1 Diabetes

**DOI:** 10.1155/pedi/5053872

**Published:** 2025-01-16

**Authors:** Kalyan Pamidimukkala, Michael L. Ferm, Madhav Erraguntla, Balakrishna Haridas, Achu Byju, Mark Lawley, Sruthi Menon, Carolina Villegas, Siripoom McKay, Daniel J. DeSalvo

**Affiliations:** ^1^Department of Industrial and Systems Engineering, Texas A&M University, College Station, Texas, USA; ^2^Department of Pediatrics, Baylor College of Medicine, Houston, Texas, USA; ^3^Department of Biomedical Engineering, Texas A&M University, College Station, Texas, USA

**Keywords:** continuous glucose monitoring (CGM), driving, hypoglycemia

## Abstract

**Background:** Clinical guidelines on driving for people with diabetes exist, but there are limited studies analyzing glucose data and hypoglycemia risk while driving. No published studies have analyzed teenage or emerging adult drivers with type 1 diabetes (T1D). The primary aim of our pilot study was to explore the glycemic patterns of young drivers with T1D as they relate to clinical guidelines and identify trends that could be used to improve road safety.

**Methods:** In this pilot study, we collected continuous glucose monitoring (CGM) data from five drivers with T1D (median age 19, range 17–21 years) over a 1-month period. The driving trips were divided into two categories: (1) *Short trips* (<60 min) and (2) *Long trips* (≥60 min). Hypoglycemia was defined as <70 mg/dL as recorded by CGM for at least four consecutive readings. Trips <10 min were excluded from the analysis.

**Results:** Data on 284 total trips with associated CGM readings were recorded. The average number of trips taken by drivers during the study was 56.8 trips (range 9–82). For short trips (*n* = 276), no episodes of hypoglycemia occurred when starting glucose was >90 mg/dL (*n* = 227). For short trips with starting glucose of 70–90 mg/dL (*n* = 32), each hypoglycemic event (*n* = 5) had a drop in the first CGM glucose value while driving. Seventeen (5.7%) of short trips started with a glucose <70 mg/dL. A total of eight long trips (>60 min) were recorded, all had a starting CGM value of >90 mg/dL, and none had hypoglycemia events.

**Conclusions:** These real-world findings from a small sample of teenage and young adult drivers with T1D support the American Diabetes Association (ADA) recommendation for starting glucose of >90 mg/dL when driving. Larger studies would be helpful in clearly identifying and improving road safety concerns in young drivers with T1D.

## 1. Introduction

Driving a car is a complex task, requiring cognitive and physical coordination to perform safely. Along with other chronic diseases, diabetes has been associated with an increased incidence of motor vehicle mishaps, likely due to the impact of hypoglycemia on cognitive and motor function [1–10]. Adolescents and young adults with type 1 diabetes (T1D) are a unique population in whom driving has not been directly studied. Emerging adulthood is a time of worsening glycemic control in people with T1D [11] and a high-risk period for motor vehicle crashes in teens and emerging adults [12]. Thus, driving presents an especially high risk in this vulnerable group.

To address concerns about driving with diabetes, clinical guidelines have been developed [13–15]. For people at risk of hypoglycemia, the American Diabetes Association (ADA) recommends a starting glucose of >90 mg/dL before driving. Regular glucose monitoring is recommended on trips >1 h, and if hypoglycemia occurs, the person should stop driving until the glucose has returned to normal range for >30 min [13]. Although these recommendations are based on expert consensus, limited real-world data exists to support them [5, 16, 17].

We examined the application of current diabetes driving guidelines using a study design similar to a recent study by Trawley et al. [16] which analyzed glucose data from 18 adult drivers (median age 40 years) with T1D using a blinded/masked continuous glucose monitor (CGM) over a 3-week period. Our study included two key differences with real-world factors not previously examined in the literature: (1) inclusion of only adolescents and young adults (≤21 years old) with T1D and (2) use of real-time, nonblinded CGM by participants. The primary aim of our pilot study was to explore the glycemic patterns of young drivers with T1D as they relate to clinical guidelines and identify trends that could be used to improve road safety [18–24].

## 2. Methods

These data were derived from a real-world data-gathering study aimed at developing future technologies incorporating sleep, exercise, and driving into hypoglycemia prediction models. Subjects were eligible to participate if they had a clinical diagnosis of T1D with duration >1 year and personal use of Dexcom G6 CGM (Dexcom, Inc., San Diego, CA, USA). Subjects were ineligible to participate if using an automated insulin delivery (AID) system or if hemoglobin A1c at study enrollment was >11%. The data included in this study are from five participants aged 16 years or above with a valid driver's license.

As part of the real-world data gathering study, participants had a Bluetooth FM transmitter (Model BH347A, VTIN Corporation) installed in the 12 V outlet of their cars and a mobile phone (Samsung J Series) application was developed to automatically connect to the transmitter when the car was powered on. The connection of the application to the Bluetooth transmitter indicated the start of a driving event and disconnection indicated the end of a driving event. For all trips, study participants were required to record on the cell phone app if they were driving or passengers and only driving trips with active CGM use were included in the analysis.

For this study, the trips were divided into two categories: (1) *Short trips* (trip time <60 min) and (2) *Long trips* (trip time ≥60 min). Trips <10 min were excluded from the analysis. Hypoglycemia was defined as sensor glucose <70 mg/dL as recorded by the CGM for at least four consecutive readings.

## 3. Results

The five drivers in the study recorded a total of 284 trips longer than 10 min that were included in the analysis. Three participants were female, two were male, and the median age was 19 years (range 17–21 years). Clinical and demographic data for each of the five drivers are presented in Table 1. All four participants on insulin pumps used a tubeless Omnipod Eros or DASH Pod (Insulet Corporation, Acton, MA, USA) without predictive low glucose suspend or closed-loop algorithm. The average number of trips taken by drivers during the study was 56.8 trips (range 9–82). The median trip time was 21.6 min (range 10.1–133.4 min).

Among 227 short trips recorded with a starting glucose >90 mg/dL, no episodes of hypoglycemia occurred. There were 32 trips with a starting glucose between 70 and 90 mg/dL and 5 of these trips (15.6%) involved hypoglycemia. Seventeen (5.7%) of short trips started with glucose <70 mg/dL, though in most of these trips (58.8%) glucose values increased within 10 min of starting the trip. The percent of glucose values below the range (<70 mg/dL) based on starting glucose range is displayed in Table 2.

A total of eight long trips (>60 min) were recorded, and all had a starting CGM value of >90 mg/dL. None of these long trips had hypoglycemia events. The average glucose rate of change was −0.395 ± 0.57 mg/dL/min for short trips and −0.89 ± 0.25 mg/dL/min for long trips. Among all trips with initial glucose >90 mg/dL, the average glucose rate of change was −0.445 ± 0.595 mg/dL/min. For trips with initial glucose of 70–90 mg/dL, the average glucose rate of change was −0.25 ± 0.339 mg/dL/min, and for trips with initial glucose <70 mg/dL, the average glucose rate of change was 0.188 ± 0.23 mg/dL/min.

**Figure 1:**
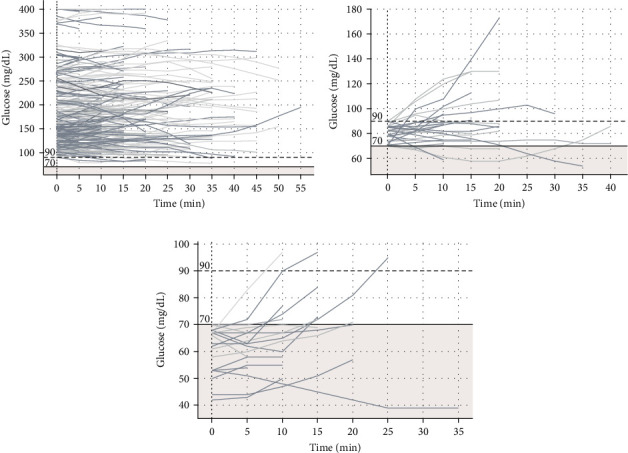
Plots of CGM values during drives. (A) Trips with initial glucose >90 mg/dL. (B) Trips with initial glucose of 70–90 mg/dL. (C) Trips with initial glucose <70 mg/dL. CGM, continuous glucose monitoring.

The plots of CGM values over time during recorded drives for trips with initial glucose >90, 70–90, and <70 mg/dL are presented in Figure 1.

## 4. Discussion

To our knowledge, this is the first study reporting on real-time CGM glucose patterns in teenagers and young adults with T1D while driving. The ADA guidelines [13] on driving with diabetes present multiple recommendations that our study explored. The ADA recommends a glucose >90 mg/dL before starting to drive. We found that no hypoglycemia events occurred among all drives with initial glucose >90 mg/dL. Similarly, in a previous study of 18 adult drivers with T1D who completed 475 rides in a 3-week period, there were no hypoglycemia events for drives that started with glucose >90 mg/dL [16]. Thus, the ADA recommendation for starting glucose >90 mg/dL in drivers with T1D appears to be supported by clinical evidence in teenage, young adult, and older adult drivers.

For starting glucose of 70–90 mg/dL before driving, the ADA recommends consuming a carbohydrate-containing snack to prevent hypoglycemia [13]. However, prior survey data [25] indicated that 36% of adult drivers with T1D had experienced hypoglycemia while driving in the preceding year [25]. Teenage and young adult drivers in our study did occasionally experience hypoglycemia when starting drives in the range of 70–90 mg/dL, and it's possible that consuming carbohydrates prior to driving may have prevented hypoglycemia. Despite the inherent risks of driving while hypoglycemic, 5.98% of trips in our study started with CGM value <70 mg/dL. Fortunately, there were no reported collisions, driving mishaps, or other events related to cognitive functioning and driver safety in the context of hypoglycemia. For the trips that started with glucose <70 mg/dL in our study, most (58.8%) had glucose values increase within 10 min of the trip. This rise in glucose could indicate that the driver appropriately consumed fast-acting carbohydrates before starting to drive with hypoglycemia; however, causality cannot be determined as multiple factors that were not obtained in the study could have affected sensor glucose readings including timing of last meal, sensor location [26], or pressure on sensor leading to compression artifact [27] while driving. The ADA provides guidance to wait for 30–60 min after treating hypoglycemia before driving, which is based on previous studies showing decreased cognitive function and decision-making even after resolution of hypoglycemia [2, 3], though studies have found the effect of prior hypoglycemia on driving ability to be variable [5, 6]. Thus, further research is warranted to determine a safe amount of time to wait before resuming driving after mild hypoglycemia occurs [6].

The ADA also recommends that glucose should be checked regularly on trips longer than 1 h [13]. A 2002 simulation study using a fixed insulin and variable glucose infusion model demonstrated that a higher glucose infusion rate was necessary to maintain euglycemia while using a driving simulator when compared to passively observing the simulation [28]. Analyses of metabolism data [29] have also indicated that driving requires a similar energy expenditure as easy walking. Our data and these prior studies support the need for real-time glucose monitoring and consumption of carbohydrates as needed to avoid hypoglycemia on longer trips.

Hypoglycemia-induced neuroglycopenia disrupts cognitive-motor functioning, which can cause an increased risk for motor vehicle accident. In a prospective study of 452 adults with T1D examining the occurrence of neuroglycopenia-related driving impairments over a 1-year period, 52% of drivers reported at least one hypoglycemia-related driving mishap, 32% reported two or more, and 5% reported six or more including collisions, citations, losing control, or someone else taking over while driving. Most driving mishaps (78%) occurred when glucose was ≤90 mg/dL within 30 min of initiating their drive, but only 35% of mishaps had an associated glucose value recorded since the study participants were using traditional blood glucose meters rather than CGM [17]. Our study is the first to observe drivers with T1D using real-time, nonblinded CGM. Past studies assessing driving in T1D [5] have used blinded CGMs without real-time alerts and glucose data were only able to be reviewed by the researchers retrospectively. With traditional blood glucose meters, measuring glucose is a two-handed process that would be unsafe to perform while actively driving. By contrast, the use of real-time CGM allows for hands-free, audio-based alerts that can be used while driving to be alerted to glucose excursions in the hypoglycemia range. While it is still recommended to stop driving if glucose value drops below 70 mg/dL, CGM alerts and trend arrows could potentially alert the driver to preventatively consume a carbohydrate-containing snack in advance of hypoglycemia. Additionally, there is potential to use predictive analytics-based methods [30–32] to improve hypoglycemia detection while driving. AID systems with the ability to attenuate and suspend insulin for down-trending glucose could further mitigate hypoglycemia risk while driving.

There were several significant limitations to our study, most notably the small sample size with a broad range of total trips taken by each driver, which prevents drawing causality relationships. Furthermore, only limited inferences can be drawn from long trip data given the small number of these trips. This pilot study did not include food intake or insulin delivery data, which restricted the ability to understand the cause of hypoglycemia episodes while driving. Given that CGM data was available in real-time to the drivers, the findings from our study may not be applicable to people with T1D using intermittent scan CGM or self-monitoring of blood glucose. Another important limitation is that the use of closed-loop, AID systems precluded participation in the larger parent study designed to develop a hypoglycemia prediction algorithm [30, 31]. AID systems are now widely available and should be included in future T1D driving studies, especially considering their potential to minimize hypoglycemia. Young T1D drivers with A1c >11% were excluded due to safety concerns, which limits the generalizability of our findings. To address these notable gaps, future research should include adequately powered samples of young T1D drivers using various insulin delivery modalities including AID systems with detailed food logs to enable in-depth analysis of glucose patterns while driving and causal relationships of glucose excursions and any potential collisions, mishaps, or other events related to cognitive functioning and driving safety.

## 5. Conclusions

Real-world data from this small pilot study suggest that current ADA guidelines on diabetes management during driving are appropriate for teens and young adults with T1D including the recommendation for a starting glucose value >90 mg/dL to avoid hypoglycemia while driving. Larger studies would be helpful in clearly identifying and improving road safety concerns in young drivers with T1D [33]. Future research should explore the effectiveness of AID systems in reducing hypoglycemia risk while driving with T1D.

## Figures and Tables

**Table 1:** Characteristics of the teenage and young adult drivers with T1D.

Participant	Age (years)	Sex	T1D duration (years)	Baseline HbA1c	Treatment regimen	Total short trips (*n*)	Total long trips (*n*)
1	18	M	3.67	6.7	SAP	80	2
2	20	F	2.25	6.4	SAP	71	1
3	21	F	9.5	8.4	SAP	67	4
4	19	F	2.17	6.5	SAP	8	1
5	17	M	2.92	9.3	MDI	50	0

Abbreviations: MDI, multiple daily injections; SAP, sensor augmented pump; T1D, type 1 diabetes.

**Table 2:** Percent of time below range (<70 mg/dL) based on starting glucose value for short trips.

Initial CGM glucose value	Number of trips	Percent of time below range (<70 mg/dL) during the trip (%)
<70 mg/dL	17	76.5
70–90 mg/dL	32	23.6
>90 mg/dL	227	0

Abbreviation: CGM, continuous glucose monitoring.

## Data Availability

The data can be made available upon request by emailing the contact author, Dr. Daniel DeSalvo at desalvo@bcm.edu.
